# Cognition and BDNF levels in prediabetes and diabetes: A mediation analysis of a cross-sectional study

**DOI:** 10.3389/fendo.2023.1120127

**Published:** 2023-03-02

**Authors:** Betul Sumbul-Sekerci, Abdusselam Sekerci, Ozge Pasin, Ezgi Durmus, Zeynep Irem Yuksel-Salduz

**Affiliations:** ^1^ Department of Clinical Pharmacy, Faculty of Pharmacy, Bezmialem Vakif University, Istanbul, Türkiye; ^2^ Department of Internal Medicine, Faculty of Medicine, Bezmialem Vakif University, Istanbul, Türkiye; ^3^ Department of Biostatistics, Faculty of Medicine, Bezmialem Vakif University, Istanbul, Türkiye; ^4^ Department of Medical Biochemistry, Bezmialem Vakif University, Istanbul, Türkiye; ^5^ Health Sciences Institute, Bezmialem Vakif University, Istanbul, Türkiye; ^6^ Department of Family Medicine, Faculty of Medicine, Bezmialem Vakif University, Istanbul, Türkiye

**Keywords:** diabetes, cognitive impairment, BDNF, mediation analysis, prediabetes

## Abstract

**Aim:**

Clinical and epidemiological studies suggest links between dementias and Type 2 diabetes (T2DM). The underlying mechanisms of diabetes-related cognitive impairment are largely unknown. This study aims to investigate the role of BDNF in cognitive impairment in prediabetes and T2DM.

**Methods:**

The study included 68 patients with prediabetes (preDM), 96 patients with T2DM, and 65 healthy controls. The cognitive function of the patients was evaluated with the Montreal Cognitive Assessment (MoCA) test and serum BDNF levels were measured by Elisa. The MoCA scores and BDNF levels were compared between diabetes groups after adjusting for age, gender, and education using ANCOVA. The role of BDNF in the diabetes-related cognitive impairment was investigated through mediation analysis.

**Results:**

Patients with T2DM had significantly lower cognitive performance, particularly in memory. Diabetes was found to be a predictor of both cognitive impairment and BDNF levels. A significant increase in serum BDNF levels was observed in patients with T2DM. However, the mediator role of BDNF in the pathology of cognitive impairment in diabetes was not determined.

**Conclusion:**

Cognitive impairment is prevalent in patients with T2DM and should be included in routine screening for complications. The results of the mediation analysis suggest that although BDNF is a biomarker affected by T2DM and cognition, it does not play a mediator role between cognitive impairment and diabetes.

## Introduction

Cognitive impairment is increasingly described as a considerable comorbidity and complication of type 2 diabetes mellitus (T2DM) (1). With the rising incidence of diabetes and longer life expectancy of T2DM patients, it is crucial to recognize cognitive problems and related factors in these individuals (2). Cognitive impairment can occur at any age and can range from mild cognitive decline to dementia (3). The relationship between prediabetes and cognitive impairment is unclear, as some studies suggest a connection (4–7) while others do not find a relationship (8).

Clinical and epidemiological studies suggest links between Alzheimer’s disease (AD), vascular dementia, and T2DM. However, the underlying mechanisms and causality between diabetes and specific cerebral changes are not well understood (9). Despite strong epidemiological evidence linking diabetes to increased risk of dementia, the neuropathology of AD does not appear to be higher in T2DM patients (10, 11). Furthermore, while diabetes is linked to an increased risk of stroke and cerebrovascular disease, this does not appear to be the major cause of the higher risk of dementia (12). Cognitive impairment in diabetes is believed to result from complex pathological processes influenced by multiple factors, some of which are unique to diabetes (13).

Critical regions of the central nervous system have high insulin receptor expression, and impaired insulin signaling in the brain can result in neuronal and synaptic loss and cognitive impairments. The number of studies looking into potential biomarkers for understanding changes in the brains of T2DM patients is rapidly expanding. Numerous animal studies suggest that BDNF may be one of the molecular factors linked to cognitive neuropathology in T2DM (14). Neuroimaging has shown that elevated glucose levels are correlated with cognitive decline and decreased hippocampal volume, serving as an early marker of diabetes-related brain damage (15). Given the high expression of BDNF in hippocampus neurons, it may be a useful early marker of cognitive impairment in diabetes.

BDNF, one of the neurotrophin family proteins that stimulates neuron regeneration and is associated with plasticity, is also responsible for energy homeostasis and is also associated with glucose metabolism and insulin resistance (16). BDNF is crucial for learning and memory processes, as it drives long-term potentiation in the hippocampus and synaptic changes. Altered BDNF levels have been associated with increased appetite, obesity, type 2 diabetes, schizophrenia, depression, and neurodegenerative diseases such as Alzheimer’s (17–20). Although BDNF’s role in glucose metabolism is significant, the exact mechanism is not known (21). While there were different results between serum BDNF levels and glucose in T2DM; no association was found with insulin (17).

This study aims to examine cognitive performance and related variables in individuals with prediabetes and diabetes. BDNF, which has links to both neuron regeneration and glucose metabolism, may play a crucial role in the cognitive impairment associated with diabetes. The primary goal of this study is to look into the probable function of BDNF in diabetes-related cognitive impairment in both prediabetes and diabetes using mediator analysis.

## Materials and methods

### Participants and sample size

This research was designed as a observational cross-sectional study. At 95% confidence level, with 80% power, when the effect size is 0.25 (medium effect) for the ANCOVA model, the degree of freedom for the main group variables is 2, the number of groups (gender*diabetes group= 2*3 = 6) is 6 and the number of covariates (age*education years) is 2, a minimum of 158 people in total should be included in the study. The sample size was calculated by G-Power (Version 3.1.9.7).

A total of 229 volunteers who applied to outpatient clinic of Internal Medicine Department, Bezmialem Vakif University Faculty of Medicine were enrolled in this study between August 2021 and August 2022. T2DM and prediabetes diagnoses of the patients were evaluated by the internal medicine specialist according to the ADA (American Diabetes Association) 2021 criteria (22). The inclusion criteria were as follows: (a) age from 30 to 65 years; (b) at least primary school education. The exclusion criteria were as follows: (a) severe psychiatric (e.g,major depressive disorder) or neurologic disorder (demyelinating diseases, stroke or brain tumor) (c) insulin use (Insulin therapy was taken as an exclusion criterion to avoid the effect of hypoglycemia on cognition) (d) vision and hearing problem (e) Hypothyroidism, B12 and folic acid deficiency (f) Advanced chronic renal failure (stage 4-5) (g) uncontrolled hypertension (h) patients with hypoglycemia-hyperglycemia attacks (h) Alcohol, substance addiction. All participants signed written informed consent, and approval was obtained from the Bezmialem Vakif University Clinical Research Ethics Committee (28.07.2021-12/1). (Clinical Trial Registration Number: NCT05654727)

Demographic data of the patients, smoking and alcohol use, and the the medications they were taking were collected. The patients’ physical activity levels were evaluated using the “International Physical Activity Scale” (23) as it may have an effect on BDNF.

### Cognitive assessment

The cognitive performance of the participants was evaluated using the Montreal Cognitive Assessment (MOCA) Test (24). The MOCA Test evaluates various cognitive functions such as visuospatial-executive functions, naming, memory, attention, language, abstraction, and orientation over a total of 30 points. For participants with 12 or fewer years of education, 1 point was added to their total score to account for their lower education level. Based on a validation study conducted with the Turkish population (25), a score of 21 or higher was considered to indicate normal cognition (NC), while a score below 21 was classified as impaired cognition (IC).

### Laboratory

Blood samples were collected from patients and controls after an 8-hour fast in the morning. The samples were placed in gel separation tubes, centrifuged at 3500xg for 10 minutes at room temperature, and stored at -80°C until the study was completed. Serum BDNF levels were determined by using a sandwich-enzyme-linked immunosorbent assay (ELISA) with the Elabscience Human BDNF Elisa Kit (Cat.No.:E-EL-H0010). The results are presented in pg/mL.

In addition to measuring serum BDNF levels, routine laboratory methods were used to measure fasting serum glucose, Hemoglobin A1c, OGTT, insulin, HOMA-IR, LDL, HDL, triglycerides, TSH, B12, folic acid, and hemogram at Bezmialem Vakif University Hospital.

### Statistical analysis

The descriptive statistics for the qualitative variables in the study were presented as numbers and percentages, while the quantitative variables were presented as mean, standard deviation, median, minimum, and maximum. The relationships between qualitative variables were examined using Pearson’s chi-square test and Fisher-Freeman-Halton tests. Normal distribution assumption was investigated using the Shapiro Wilk test. The independent sample t test (student t test) was used to compare the mean of two independent groups, and the Mann Whitney U test was used to compare the of the medians of two independent groups. One-way analysis of variance (one-way anova) was used for the mean comparison of more than two independent groups, and the LSD method was used for multiple comparisons. Kruskall-Wallis test was used in comparison of the medians of more than two independent groups, and Dunn’s test was used as a *post hoc* test in pairwise comparisons. Relationships between quantitative variables were investigated with Pearson and Spearman correlation coefficients. ANCOVA analysis was performed to eliminate the effects of covariate and factors, and mean and standard deviation values were given with corrections. Variables with a p value of up to 0.25 in univariate analyzes were included in the model in multivariate analysis. Linear regression analysis were used when the dependent variable was quantitative. The stepwise method was used as the variable selection method. In addition, mediation analysis, which is a regression-based path analysis technique, was performed to examine the effect of BDNF. Hayes’ PROCESS macro was used in mediation analysis. Ordinary least-squares framework was used to estimate the direct and indirect effects in the method. The significance of the effects was also evaluated with the Sobel test. The statistical significance level was taken as 0.05, and the SPSS (version 26, Chicago, IL, USA) package program was used in the calculations.

## Results

A total of 229 participants (68 with preDM, 96 T2DM and 65 controls) were recruited (mean age 49.98 ± 7.62 years, 57.6% women). The clinical and demographic characteristics of the participants are presented in **Table 1**.

**Table 1:** Demographic and clinical characteristics of the participants.

Variable	PreDM (n:68)	DM (n:96)	Control (n:65)	p	*Post hoc* p
Age	51.28 (7)	49.96 (8.38)	47.88 (6.07)	**0.015**	PreDM-C=0,004
Female, n(%)	50 (73.5)	39 (40.6)	43 (66.2)	**<0.001**	
Education,years	7.03 (2.89)	7.76 (3.64)	9.22 (3.83)	**0.008**	PreDM-C=0.002 DM-C=0.019
BMI	29,95 (4.56)	30.40 (4.60)	27.84 (4.83)	**0.012**	PreDM-C=0,025 DM-C=0,004
Fasting Glucose (mg/dL)	103 (83-122)	139 (22-381)	92 (70- 99)	**<0,001**	PreDM-DM<0,001 PreDM-C=0,001 DM-C<0,001
Hemoglobin A1c (%)	5.7 (5-6.40)	7.23 (4.67-13.20)	5.41 (4.63-5.69)	**<0,001**	PreDM-DM<0,001 PreDM-C =0,001 DM-C<0,001
OGTT	121,98(83-199)	227,87 (86-590)	102 (64-138)	**<0,001**	PreDM-DM<0,001 PreDM-C =0,009 DM-C<0,001
Insulin	9.63 (3.86)	8.53 (3.23)	8.24 (3.88)	0,212	
HOMA-IR	2.44 (0.62- 98)	3.05 (1.56-6.50)	1.61 (0.68-4.44)	**<0,001**	PreDM-DM=0,013 PreDM-C=0,007 DM-C<0,001
LDL (mg/dL)	138.91 (39.28)	136.9 (33.75)	128.61 (28.91)	0,268	
HDL (mg/dL)	53.04 (13.45)	47.83 (13.07)	53.4 (12.94)	0.106	
Triglycerides (mg/dL)	133 (24-387)	149 (55-1034)	113 (44-405)	**<0,001**	PreDM-DM=0,046 DM-C<0,001
TSH	1.76 (0.09-5.84)	1.71 (0.52- 6.02)	1.5 (0.32-4.5)	0,369	
B12 (pg/mL)	296 (168-2000)	284 (135-899)	306 (112-2000)	0.975	
Folic Acid	6.6 (2.7-20.4)	7.94 (2.5-17.8)	7 (3.6-12.20)	0,137	
25(OH)D (ng/mL)	16.20 (7.97)	12.3 (7.87-22.25)	13.07 (3.25-35.40)	0.573	
Neutrophil (10*3/uL)	55,80 (6.98)	54,87 (7.38)	54,90 (7.28)	**0.033**	DM-C=0,009
Lymphocyte (10*3/uL)	2.42 (0.71)	2.75 (0.83)	2.37 (0.55)	0.004	PreDM-DM=0,009 DM-C=0,003
NLR	1.72 (0.71-3.47)	1.57 (0.68- 3.17)	1.56 (0.84-11.04)	0.651	
PLR	107.9 (33.25)	98.73 (25.79)	112.65 (30.46)	**0.013**	PreDM-DM=0,076 DM-C=0,004
Hemoglobin	13.64 (1.49)	14.41 (1.53)	13.44 (1.59)	**<0,001**	PreDM-DM=0,005 DM-C<0,001
Platelet	246.33 (64.03)	255.73 (63.95)	262.16 (65.91)	0.415	
Smoker, n (%)	21 (32)	23 (24)	13 (21)	0.436	
Alcohol user, n (%)	5 (7)	14 (15)	4 (7)	0.250	
Physical activity level				0.311	
inactive	46 (68)	66 (69)	43 (66)		
minimally active	18 (26)	30 (31)	21 (32)		
sufficiently active	4 (6)	–	2 (2)		

Normally distributed continuous variables were presented as mean ± SD, non-normally distributed continuous variables were presented as median (Q1-Q3), Categorical variables were presented as counts (percentages) NLR, Neutrophil-lymphocyte ratio; PLR, Platelet-lymphocyte ratio. Statistically significant values are written in bold.

### Cognition in patients with prediabetes and T2DM

The patients were divided into two groups, impaired cognition (IC) and normal (NC), based on their MoCA scores. The prevalence of cognitive impairment was 27.3% in the preDM group, 34.7% in the DM group, and 10% in the control group. The incidence of impaired cognition was significantly higher in the T2DM group compared to controls (chi-square= 7.486 p=0.024).

MoCA total score and subscores were compared between diabetes groups by adjusted for age, gender, and education with ANCOVA. The mean MoCA total score for the T2DM group was 22.89 (SD 3.71), while it was 24.95 (SD 3.25) for the control group (adjusted means for age, gender, and education). A significant decrease in cognitive performance was observed in patients with T2DM compared to healthy volunteers (p=0.01). The memory performances of patients with diabetes were significantly impaired compared to healthy volunteers (p=0.031) (**Table 2**).

**Table 2:** Comparison of total score and subscores of MOCA between patients with prediabetes, diabetes and healthy controls.

Cognition	PreDM	DM	Control	p-value
MoCA Total score	22.89 ± 3.71	22.48 ± 4.08*	24.95 ± 3.25	**0.022**
Visuospatial/Executive	3.64 ± 1.00	3.59 ± 1.07	4.10 ± 1.00	0.176
Naming	2.51 ± 0.50	2.49 ± 0.60	2.68 ± 0.61	0.381
Attention	4.31 ± 1.35	4.72 ± 1.46	4.88 ± 1.26	0.983
Language	1.47 ± 0.97	1.29 ± 1.08	1.68 ± 0.91	0.165
Abstraction	1.47 ± 0.60	1.29 ± 0.76	1.63 ± 0.58	0.083
Memory	2.82 ± 1.37	2.48 ± 1.36*	3.38 ± 1.38	**0.031**
Orientation	5.71 ± 0.53	5.79 ± 0.47	5.87 ± 0.33	0.589

Data presented as mean ± standard deviation. Values in the table have been adjusted for age, gender, and education years with ANCOVA.

* indicates significance (p ≤ 0.01) of the comparisons between healthy controls and T2DM. Statistically significant values are written in bold.

The mean MoCA score of preDM patients was 22.89 (SD 3.71). Similar to the T2DM group, a decline in cognitive performance was observed in patients with PreDM. When comparing the MoCA total and sub-scores of the diabetes groups with ANOVA, cognitive performance was significantly impaired in both the PreDM (p=0.01) and T2DM groups (p=0.001). However, after adjusting for age, gender, and education, no significant difference was found between the preDM and control groups (**Table 2**).

### Serum BDNF levels in diabetes and cognition groups

**Figure 1:**
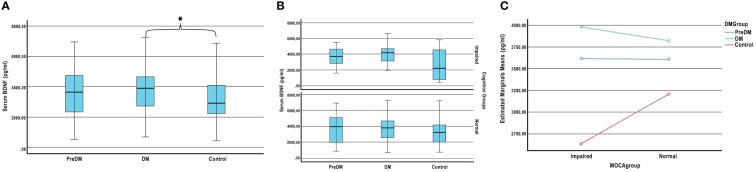
Plasma BDNF levels according to the stages of the diabetes and cognition. **(A)** Comparison of BDNF levels between diabetes group. **(B)** Comparison of BDNF levels between diabetes and cognitive groups. **(C)** Effect of diabetes on plasma BDNF levels according to cognitive stages. ANCOVA adjusted for age, sex, and education; * *P* < 0.05.

Serum BDNF levels of the patients were compared between diabetes groups (T2DM, PreDM and Control) and cognitive groups (IC-NC). While the mean BDNF level of the DM group was 3854.71 ± 1492.18 pg/mL, it was 3131.23 ± 1548.94 pg/mL for the control group (adjusted for age, gender, and education). A significant increase was observed in the serum BDNF level of patients with T2DM compared to healthy volunteers (p=0.05). The mean BDNF level of the preDM group was 3667.81 ± 1543.86 pg/mL, no significant difference was seen compared to healthy volunteers (p=0.162). (**Figure 1A**).

There was no significant difference in BDNF levels between the cognition impaired and normal groups (p=0.357). Also the BDNF levels of patients with IC and NC in the preDM, DM and control groups were compared. Although there was no significant difference between the groups (respectively p=0.45, p=0.248, p=0.295) (**Figure 1B**), BDNF distribution pattern was different in diabetes groups according to cognition (**Figure 1C**). The mean BDNF levels of impaired cognition and normal cognition in the PreDM group were very close. However, while BDNF levels of impaired cognition group was higher in the DM group, it was lower in healthy volunteers.

### Relations between BDNF, cognitive performance, clinical profiles and diabetes

A significant correlation was observed between Moca total score and age (r= -0.240, p=0.002), education (r=0.492, p<0.001), disease duration (only for T2DM patients; r=- 0.266, p=0.032), fasting glucose and Hba1c (r=0.-239, p=0.002) variables. In the regression model, diabetes group and education were predictive variables of MoCA score was detected (**Table 3**). It was determined that being in the T2DM group decreased the MoCA score by 1.2 times, and an increase in education level increased it 0.5 times.

**Table 3:** Linear regression analysis for MoCA and BDNF score.

		B	Std. Error	p	95,0% CI for B	
**BDNF**	**(Constant)**	2805,785	545,743	<,001	1726,473	3885,097
	**Platelet**	5,811	1,887	,003	2,079	9,542
	**Control Group**	-607,110	301,921	,046	-1204,216	-10,003
**MoCA**	**(Constant)**	19,685	,852	<,001	17,998	21,372
	**Education year**	,538	,088	<,001	,365	,712
	**T2DM Group**	-1,298	,628	,041	-2,542	-,055

The correlation of BDNF score with platelet level (r=0.141, p=0.05) and memory (r=-0.172, p=0.031) was significant. In linear regression analysis, diabetes and platelet variables were found to be significant in predicting BDNF level (**Table 3**). Interestingly, our analysis showed that increase in platelet level was a significant predictor of increased serum BDNF levels.

### Investigation of the mediator role of BDNF between diabetes and cognitive impairment

**Figure 2:**
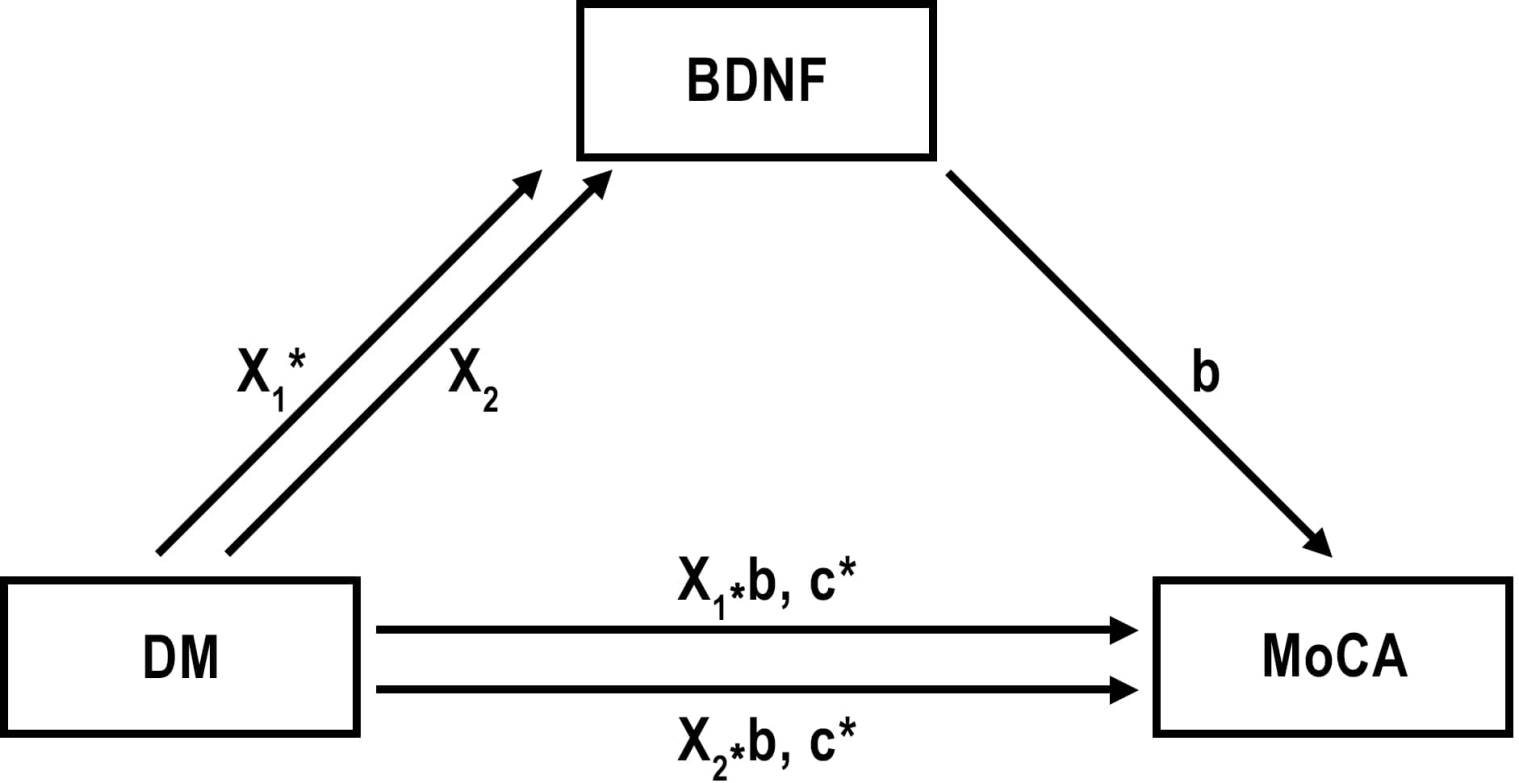
Effect of diabetes and prediabetes on MoCA through BDNF. X_1_= Effect of T2DM on BDNF; X_2_= Effect of preDM on BDNF; b= Effect of BDNF on MoCA; X_1*_b and X_2*_b are indirect effects of DM and PreDM on MoCA. c= direct effect of DM on MoCA. *p ≤ 0.01.

We hypothesized that BDNF levels may mediate to explore or understand the underlying mechanism in the cognitive impairment in diabetes. BDNF levels were affected by both diabetes pathology and cognitive processes in our patients. The mediator role of BDNF in the relationship between diabetes and cognitive impairment was investigated by mediation analysis. We had 3 groups (DM-PreDM-C) in the diabetes group. Dummy coding was done for the diabetes groups variable in order to perform mediator analysis. In coding, the control group was taken as the reference group. While the effect of DM was evaluated for X1, the effect of preDM was evaluated for x2. Standardized regression coefficient (β) for the association between DM and BDNF (X_1_ in the **Figure 2**) was 784.58 (p=0.013) and it was 561.95 (p=0.103) between preDM and BDNF (X_2_ in the **Figure 2**). These findings show that the change in BDNF levels is significant for the control and DM groups, and insignificant for the control and preDM groups. The standardized regression coefficient (β) for the association between BDNF and MoCA (b) was -0.0002 (p=0.404) (**Figure 2**). As BDNF goes up, MoCA goes down but it is not significant. Relative direct effects of diabetes on MoCA was -2,3143 (p=0.021) for T2DM and it was -2,1912 (0.054) for preDM. Relative total effects of diabetes on MoCA was -2.4384 (p=0.001) for T2DM and it was -2.2800 (p=0.0031) for preDM. In a model with BDNF as a potential mediator, we found that total and direct effects of diabetes on MoCA were significant for PreDM and T2DM (p<0.05). But we didn’t find a significant indirect effect of DM on MoCA through BDNF (for X_1_ Sobel test p=0.460; for X_2_ Sobel test p=0.484).

## Discussion

This study was the first to examine the role of BDNF in cognitive impairment in T2DM and prediabetes using mediation analysis. The key findings were: (a) Cognitive performance was significantly impaired in T2DM, with memory functions being the most affected. (b) Patients with T2DM showed a significant increase in serum BDNF levels. The pattern of BDNF changes varied between T2DM, prediabetes, and control groups depending on cognitive impairment. (c) T2DM was found to be a predictor of both cognition and BDNF levels. (d) The mediating role of BDNF in the development of cognitive impairment in diabetes remains to be determined.

Cognitive impairment, ranging from mild cognitive impairment to dementia, is increasingly recognized as a common complication and comorbidity in T2DM (3, 26, 27). Our study found that cognitive performance, particularly memory scores, was significantly lower in diabetes patients. The diagnosis of diabetes was found to be a significant predictor of MoCA scores. Results from the ENDBID study, similar to our findings, showed that middle-aged individuals with T2DM performed worse on the MOCA test and had lower memory scores (4). There is consistent evidence that T2DM is a separate risk factor for cognitive dysfunction in the elderly (28). These findings are important to consider in clinical practice and diabetes-related cognitive impairment should be given more attention as a complication in current guidelines, with regular screening of patients with diabetes.

In our study, similar to the T2DM group, decreased cognitive performance was observed in individuals with prediabetes. However, after adjusting for age, education, and gender, the statistical significance disappeared. The research evaluating cognitive function in prediabetes is limited and inconsistent. One systematic review (28) suggest there is insufficient evidence to support a connection between prediabetes and cognitive impairment, while another meta-analysis reported that there was moderate-to-high-quality evidence of a positive association between prediabetes and dementia (29). An 8-year longitudinal study also found no significant association between baseline prediabetes status and later cognitive function (30). Further research with larger sample sizes is needed to better understand cognitive impairment in prediabetes.

BDNF is an important protein involved in both peripheral metabolic activities and central neuronal regeneration and plasticity (18). Literature suggests that physical activity improves peripheral bdnf levels and cognition. Therefore, we also evaluated the physical activities of the patients. We did not find a significant difference between the physical activity scores of the groups (p=0.311) (**Table 1**). Studies have investigated the relationship between BDNF levels and glycemic parameters, but the results are conflicting. Some studies reported an increase in BDNF levels in T2DM patients (31, 32) while others reported a decrease (17, 33, 34). Increased BDNF levels have been reported in patients with T2DM receiving metformin therapy (35). In our study, BDNF levels were significantly higher in T2DM patients, which may be due to 90% of T2DM patients receiving metformin therapy alone or in combination. We also observed different distributions of BDNF patterns among diabetes groups in relation to cognition. In the control group, there was a positive correlation between cognition and BDNF level, whereas the relationship in T2DM was negative. Could this be considered a compensatory neuroprotective mechanism for cognitive impairment in T2DM? The differences in BDNF levels in T2DM patients in the literature may be due to the heterogeneity of cognition and antidiabetic treatment in the populations.

Because the pathways causing cognitive impairment in T2DM are unclear, it is important to identify a biomarker that may provide a potential link in the pathophysiology. Based on the data that T2DM is a predictor of both cognition and BDNF levels, we hypothesized that BDNF may play a role in diabetes-related cognitive impairment. The relationship between BDNF concentration and cognitive performance has been described in several neurodegenerative and psychiatric disorders (36). In limited studies on cognition and BDNF in T2DM patients, low BDNF levels were found to be associated with cognitive impairment and dementia (37–40). However, no study was found that investigated the relationship between cognition and BDNF in prediabetes patients. Our study’s mediation analysis suggests that although BDNF is a biomarker influenced by T2DM and cognition, it does not mediate the relationship between cognitive impairment and diabetes. Although BDNF does not have a causative role in the pathology, it may serve as a useful biomarker for monitoring cognitive impairment due to diabetes. In addition to diabetes, platelet levels have been determined as a predictive factor for BDNF. Peripheral BDNF is stored in large amounts in platelets (41), and serum BDNF levels are elevated in T2DM patients with high platelet reactivity (35).

Several limitations of this study should be noted. While research has shown that BDNF crosses the blood-brain barrier and that there is a correlation between brain and peripheral levels (42, 43) it may not be enough to fully understand the role of BDNF in neuropathology caused by diabetes. Secondly, grouping patients based on drug treatment would have allowed for observation of the effect of antidiabetic treatment on BDNF levels, but since most of our patients took metformin, this variable could not be evaluated. Lastly, a larger sample size may provide clearer results, particularly for the prediabetes group.

## Conclusion

In conclusion, the study found that T2DM patients have notable cognitive impairment, particularly in memory performance, which highlights the importance of cognitive screening for the follow-up of diabetes-related complications. The results suggest that BDNF does not play a mediatory role in the pathophysiology of cognitive impairment in T2DM.

## Data availability statement

The original contributions presented in the study are included in the article/supplementary material. Further inquiries can be directed to the corresponding author.

## Ethics statement

The studies involving human participants were reviewed and approved by Bezmialem Vakif University Clinical Research Ethics Committee (28.07.2021-12/1). The patients/participants provided their written informed consent to participate in this study.

## Author contributions

BS-S: conception and design, acquisition of data, analysis and interpretation of data, drafting of the manuscript, Final approval of the version to be published. AS: conception and design, acquisition of data, Final approval of the version to be published. OP: Statistical analysis and interpretation of data, drafting of the manuscript, Final approval of the version to be published. ED: Laboratory experiment, Final approval of the version to be published. ZY-S: Acquisition of data. All authors contributed to the article and approved the submitted version.
